# Gérard Orth: From Viral to Human Genes Underlying Warts

**DOI:** 10.1007/s10875-024-01704-x

**Published:** 2024-04-27

**Authors:** Jean-Laurent Casanova, Emmanuelle Jouanguy

**Affiliations:** 1 St. Giles Laboratory of Human Genetics of Infectious Diseases, Rockefeller Branch, The Rockefeller University, New York, NY 10065 USA; 2 Laboratory of Human Genetics of Infectious Diseases, Necker Branch, INSERM, Necker Hospital for Sick Children, 75015 Paris, France; 3 Paris Cité University, Imagine Institute, 75015 Paris, France; 4 Department of Pediatrics, Necker Hospital for Sick Children, 75015 Paris, France; 5 Howard Hughes Medical Institute, New York, NY USA

Gérard Orth was born in 1936 and died in 2023. He was picky and prickly. He was sharp and scholarly. He was stern and serious. We loved him, even when he scolded us for forgetting a footnote to an abstract for a communication at a small workshop in a tiny town in the middle of nowhere in the 1960s. Detail was everything to him, but he was also unique in his global vision, which enabled him to make biological and medical breakthroughs in rabbits and humans, but also to discover both viral and host determinants of health and disease.

At the time of his death, Gérard Orth was Emeritus Professor at the *Institut Pasteur* (where he worked from 1979 to 2003) and Emeritus Director of Research at the *Centre National de la Recherche Scientifique* (CNRS, between 1966 and 2001). He was elected to the French Veterinary Academy in 2003 and the French Academy of Sciences in 2004. Gérard worked at the *Institut Gustave Roussy* (IGR) in Villejuif from 1961 to 1979, initially in the “Laboratory of Biochemistry and Enzymology” of Claude Paoletti, and then, from 1975 onwards, in his own “Laboratory of Viral Etiologies of Human Cancers”. François Gros eventually invited him to join the *Institut Pasteur*, where he founded and led the “Papillomavirus Unit” from 1980 until his retirement in 2003. This laboratory was affiliated to the *Institut National de la Santé Et de la Recherche Médicale* (INSERM). Gérard Orth also served as Head of the Department of Virology at the *Institut Pasteur* on two occasions, between 1991 and 1996 and between 2001 and 2003.

Gérard was born to a Protestant family from Alsace. Like many Alsatians, he was a French patriot. On one occasion, on arriving for dinner, the first thing he once told us, with a certain excitement, was that he had finally understood how Joseph Meister had been able to receive Louis Pasteur’s experimental vaccine against rabies in July 1885, despite living in Alsace during the short period between the Franco-Prussian War of 1870 and World War I in which it was a German province. Gérard’s detective work revealed that Meister’s father had retained his French citizenship. That made his day. And this was typical of Gérard’s conversation, and of his life.

Gérard’s parents were born in Alsace, but they moved to Paris briefly, subsequently settling in Saint Ouen l’Aumône, a small town in *Ile-de-France* separated from the city of Pontoise by the Oise River, where there was a small Protestant community. His father worked as a manager in the agrifood industry, while his mother was a housewife. Gérard was born in Paris, 12 years after his parents’ marriage, and was an only child. He later recalled that his Alsatian and Protestant education instilled in him a “*sense of duty, responsibility, and respect for others*”.

Between 1946 and 1953, Gérard attended middle and high school at the *Lycée Jean-Claude Chabanne* in Pontoise. This school was named after a member of the Resistance from Pontoise who was murdered by the Nazis in 1942. Jacques Dupâquier (1922–2010) was the only teacher at the school he admired; he would later become an eminent historian, elected to the French Academy of Moral and Political Sciences. Gérard also took piano lessons with the famous composer Maurice Schwaab (1888–1953), who resided in Pontoise. Gérard was not gifted enough to entertain any thoughts of a musical career, but his talents were sufficient for him to play the harmonium at the Protestant church in Pontoise on Sundays. He obtained his Baccalaureate in 1953.

Gérard had considerable admiration for the civilizing and humanitarian mission of the French Empire and, at one point envisaged working in the upper echelons of administration in the French colonies. His parents opposed this plan, which, indeed, did not seem timely in 1953. So, rather than preparing for the entry examination for the *Ecole Nationale de la France d’Outre-Mer* (the National School for French Overseas Territories), he instead entered a preparatory school for veterinary training. However, tormented adolescent that he was, his chief reasons for going to veterinary school were retaliation and provocation. He hated animals and secretly hoped his parents would refuse this plan. He later admitted that this was “*an aberration for him and his parents*”. He suffered two years of intense study at the *Lycée Marcelin Berthelot* in Saint Maur des Fossés. Marcelin Berthelot was a nineteenth century French chemist and polymath. Gérard then attended the national veterinary school at Maisons-Alfort near Paris between 1955 and 1959 (Fig. 1A).

**Fig. 1:**
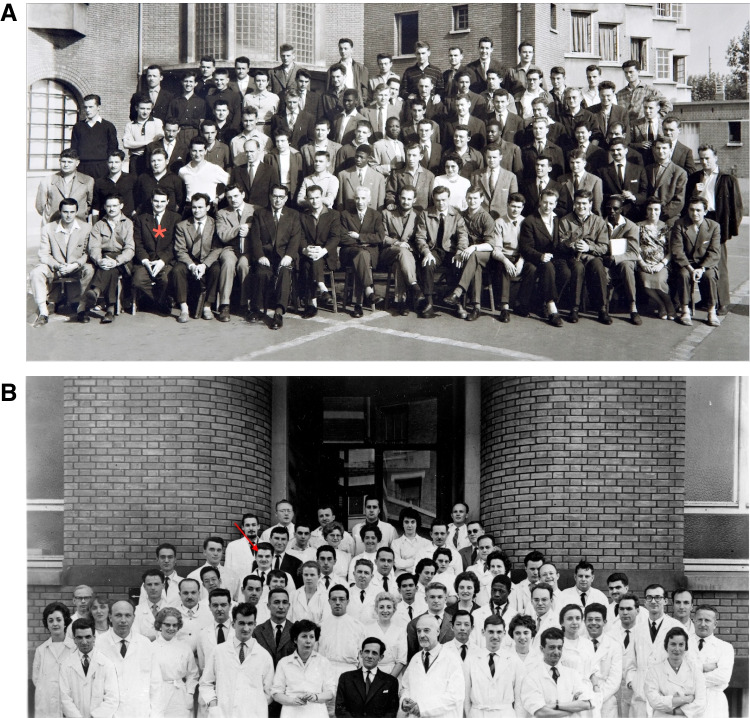
**A** 1959 promotion of the Veterinary School at Maisons-Alfort (Gérard Orth is indicated by the red cross). **B** 1959–1960 promotion of the Microbiology cours at Pasteur Institute (Gérard Orth is indicated by the red arrow)

Gérard had only a few friends among his classmates at Maisons-Alfort, one of the most important of whom was Jean-Charles Friedmann, to whom he owed his admiration for Charles de Gaulle and unreserved support for the General’s actions after his return to power. Gérard recalled that “*Charles de Gaulle was a mentor for me. He instilled in me "a certain idea of France" the roots of which I found in his writings. I experienced the death of Charles de Gaulle on November 9, 1970 as the loss of a loved one. I went to Colombey-les-deux-Eglises to attend the General’s funeral. I witnessed, by the side of the road, in the midst of thousands of the faithful, including the actor Michel Simon and the ski champion Marielle Goitschel, the departure of the armored vehicle carrying the coffin of the General from La Boisserie towards the church of Colombey. How can we forget the great distress that marked the faces of the small number of faithful, including André Malraux and Romain Gary, who were the last to pay tribute to the General before he left La Boisserie?*”.

In 1958, Gérard had attended Charles de Gaulle’s *Place de la République* speech establishing the 5th Republic, at which political opponents voiced concerns about the “fascist inclinations” of the man who had led the French resistance against the Nazis — such is the nature of French politics. Indeed, Gérard was a Gaullist, at odds with the mainstream French scientific community, which consisted essentially of supporters of various shades of communism and socialism, among whom social democrats were the rare reasonable people in the mad house. He did not really belong.

In 1957, Pierre Goret (1907–1994) welcomed Gérard to his laboratory for the preparation of his Doctorate in Veterinary Medicine (DVM), which he obtained in 1961. He later recalled that “*this status was abolished in May 1968, because it was assimilated to slavery!*”. Pierre Goret, who held the chair of contagious diseases of the Veterinary School in Maisons-Alfort, played an essential role in his career. Goret was a highly talented teacher and orator. As a tutor, he was benevolent and understanding of Gérard’s moods. With the assistance of Charles Pilet, Pierre Goret got Gérard involved in his work on an interesting topic, the then enigmatic relationship between the viruses responsible for measles in humans, distemper in dogs, and rinderpest in cattle.

Gérard’s work focused on the antigenic and immunogenic relationships between the distemper and rinderpest viruses, with the rabbit as a model. This period marks the start of his interest and expertise in rabbit biology, which later made a major contribution to his studies of papillomaviruses. The experimental approach used, based on the inoculation of rabbits with blood or tissue homogenates, with daily temperature readings and autopsies, was the one that Edmond Nocard (the discoverer of *Nocardia* and one of the famous veterinary surgeons in the Pasteur saga), the founder of the laboratory, had implemented in 1888.

This study led to his first publication as coauthor of a paper published in 1960 in the *Annales de l’Institut Pasteur* [1]*.* His DVM thesis, which he defended in 1961, was entitled "*Sur les relations antigéniques et immunogéniques du virus de la maladie de Carré et du virus de la peste bovine. Recherches expérimentales chez le lapin*" (On the antigenic and immunogenic relationships of the distemper and rinderpest viruses. Experimental studies in the rabbit) (Fig. 2). His thesis was rewarded with a prize in the form of a medal representing Henry Bouley (1814–1885), a figurehead of veterinary medicine in the nineteenth century. This inspired Gérard, who had collected stamps as a child, to collect medals, particularly those devoted to Louis Pasteur and his disciples or emulates.

**Fig. 2.**
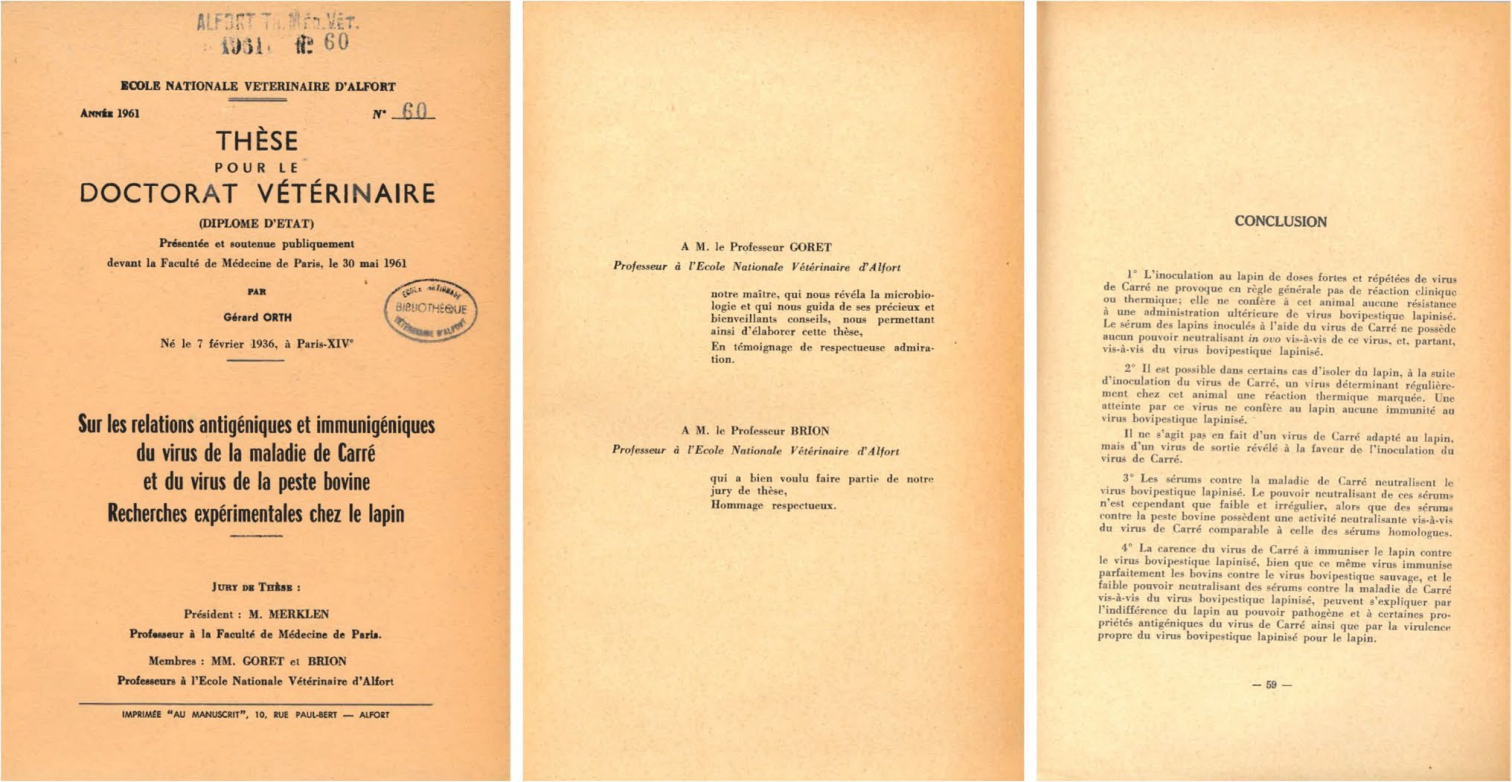
1961, Doctorate in Veterinary Medicine. Left to right: Cover page of the Doctorate in Veterinary Medicine (DVM), Acknowledgments to Profs. Goret and Brion, concluding remarks of his DVM

Goret triggered Gérard’s vocation for science and acted as his mentor from 1957 to 1961. It is to Goret that Gérard owed his attraction to microbiology and research. Goret taught him that research must have ambitious objectives and that its success is dependent on hard work and availability, including during weekends. However, Gérard rapidly came to understand that it also required a certain amount of talent and a lot of luck. From then on, his path lay clear before him: “*I will be a researcher*”. Pierre Goret was also the key to his recruitment as an agent on a scientific contract at the *Institut National de la Recherche Agronomique* (INRA) during his last year at Maisons-Alfort (1959).

Gérard was proud to be a veterinarian. He was proud that veterinarians had understood and supported Pasteur when physicians were opposed to him [2]. He was all too aware that physicians in academia were all too often more interested in power plays than in the search for truth — especially in France, where so many become addicted to “medical power”, combatting “*mandarins*” in their youth, only to become even worse “*mandarins*” as their age and power increased. He wondered how Antoine Lavoisier, who had pushed for the election of physicians to the French Academy of Science, would consider his decision with hindsight. The likes of François Magendie and Claude Bernard had become a rarity. Gérard liked to say that veterinarians were a humbler and more serious breed. He admired many, and perhaps especially Gaston Ramon (1886–1963), who had discovered the principle of vaccination with anatoxin.

Gérard attended the Microbiology (1960) and Immunology (1960) courses at the *Institut Pasteur* in Paris. The “*Grand Cours de Microbiologie*”, now defunct, was the heir to the “*Cours de Microbie Technique*” created by Pasteur in 1889 (Fig. 1B). Its primary purpose was to teach systematic microbiology. However, it also represented an opportunity, then unique in France, to discover the nascent molecular microbiology developed at the *Institut Pasteur* by André Lwoff, Jacques Monod, François Jacob, and Elie Wollman. Gérard had bought and read with great interest "*The sexuality of bacteria*", a book published by E. Wollman and F. Jacob (prefaced by A. Lwoff) in 1959, before reading Jacques Monod’s thesis on the diauxic growth of bacteria. He then “*understood the growing importance of molecular microbiology relative to traditional systematic microbiology*”.

Gérard would have to wait another 20 years to join the *Institut Pasteur*. In 1961, he met Claude Paoletti (1928–1994), who became his second mentor during his two years of military service at IGR in Villejuif, where he served as army midshipman to the naval animal facility. Paoletti headed the “Laboratory of Biochemistry and Enzymology”, which focused on the biochemistry of DNA and DNAses. He soon got Gérard involved in a new project on the biological properties of polyoma virus DNA. He encouraged Gérard to collaborate with Pierre Lépine at the *Institut Pasteur*. A collaboration was also established with Pascu Atanasiu in Lépine’s department, for the performance of tissue culture experiments that would not have been possible in Villejuif. This was Gérard’s second interaction with the *Institut Pasteur*.

Pascu Atanasiu (1913–1995) welcomed Gérard to the Virology Department (*Service des Virus*) located in the Darré Building in 1961. Gérard then discovered another facet of research. Under the auspices of Pierre Lépine, Head of Department since 1941, the writing of grant applications and activity reports was not a matter of major concern, contrary to what he had previously learnt with Paoletti. At the time, Pierre Lépine (1901–1989) personified French virology. In 1941 he was appointed head of a group of viral research departments, after the management of the *Institut Pasteur* decided to unify the various laboratories dealing with viruses into a single department. Lépine improved laboratory techniques by installing two high-speed centrifuges in his department, together with the first electron microscope in France, jointly funded by the CNRS.

Lépine participated in the drawing up of plans for the Darré Pavilion, the *Service des Virus*, at Institut Pasteur, funded by a donation from Mrs. Darré, the widow of the former chief physician of the Pasteur Hospital. Lépine’s fame was due largely to his invention of the French vaccine against poliomyelitis. In 1957, Salk and Lépine published the results of their work a few weeks apart [3–5]. The two vaccines differed in the strains used. The “Lépine vaccine”, published in 1954 [6], was produced from selected weakly neurovirulent poliovirus strains grown in the kidney cells of African monkeys not infected with the SV40 virus, which is oncogenic. Lépine prevented any risk of infection by performing a double inactivation of the virus, first with formalin, and then with beta-propiolactone. Vaccination with the Lépine vaccine was made compulsory in France in 1964, and the rapid generalization of poliomyelitis vaccination practically eliminated infantile paralysis in France.

Gérard was particularly fond of a very “French” anecdote about Lépine, who had married Marie-Madeleine Dollfus (1906–1990) in 1926. He also had a well-known, long-standing relationship with Christiane Petersen (1911–2008), with whom he had a daughter and two sons. Petersen had tried, without success, to make the Lépine’s divorce a condition for recognition of his paternity. She was behind the publication of a book by X, “*On achève bien les cobayes*”, the hero of which was inspired by P. Lépine and paints a very unflattering portrait. The sale of this book was banned until the death of P. Lépine in 1989, when both Marie-Madeleine Lépine and Christiane Petersen (both under the name of Madame Lépine!) announced his death in *Le Figaro*!

The Darré Building housed various research and diagnostic laboratories (poliomyelitis, influenza, rabies, etc.), as well as a rich library and communal laboratories for classical virology (histology, electron microscopy, preparation of sterile glassware). Unfortunately, the turning point in modern virology — molecular approaches to studying the interaction of viruses with their host cells — had not yet been reached. In the absence of seminars, the primary opportunity for interactions between members of the Virus Service was provided by attendance at the Virus Club, a restaurant located in the Borrel Building adjacent to the Darré Building, at a time when there was no cafeteria accessible to members of other departments of the *Institut Pasteur*.

Gérard later recalled “*how could I have imagined then that I would become the head of the Virology Department in 1991 and that, on my initiative, the Salle Pierre Lépine (the library of the Darré Building) would be inaugurated in 1992, decorated with a Pierre Lépine medal designed by R. Pépin? This event was commemorated by the production of a medal representing Pierre Lépine on one side and the Darré Building on the other. And how could I have imagined that both the Darré and Borrel Buildings were destined for demolition in 2024 to make way for a vector-borne infectious diseases research center!*”.

Pascu Atanasiu (1913–1995) devoted himself mainly to the study of the rabies virus. His laboratory contained a few trainees and technicians with complementary skills. He had recently become interested in tumors induced by the polyoma virus, following a stay with Karl Habel at the NIH. Atanasiu, himself of Romanian origin, had been a friend of Constantin Brancusi (1867–1957), the famous Romanian sculptor. Brancusi had made him the executor of his will, with the mission of transferring his workshop at Impasse Ronsin to the National Museum of Modern Art.

Gérard’s arrival in his laboratory coincided with the end of the fight between Atanasiu and Alexandre and Natalia Istrati, painters of Romanian origin, whom Brancusi had hosted at Impasse Ronsin, and who opposed the transfer of the studio. Gérard recalled that “*I had the privilege of attending the inauguration of the reconstitution of the studio featuring major works by the artist at the Palais de Tokyo in 1962, shortly after my arrival at the Pasteur Institute*.” Atanasiu introduced Gérard to techniques for studying the polyoma virus ex vivo and in vivo, and the collaboration between the Gustave Roussy and Pasteur institutes resulted in several publications, including Gérard’s first article (written with the help of Karl Habel) in an international journal [7].

After his military service, Gérard continued his training in genetics and biochemistry at the Faculty of Sciences (*Biochimie Générale* (1963), *Biochimie Approfondie* (1963) and *Génétique Générale* (1964) certificates) and completed his work on the polyoma virus in the context of a scheduled return to INRA*.* He presented this work at a competitive examination at the end of 1962 for the post of research assistant at the newly created Veterinary Research Department of the *IGR*. He won first place and was predicted a brilliant career as a civil servant at INRA. Paoletti then offered him a place as a PhD student studying the effects of ultraviolet irradiation on the biological properties of polyoma virus DNA in his laboratory at the IGR.

Paoletti and Orth became close friends. Gérard belonged to the “Pao Gang” and was responsible for establishing the “Claude Paoletti Prize” shortly after Paoletti’s death. One of the authors (JLC) first entered Gérard’s office in the Virology Department of the Institut Pasteur in 1997 as one of the first laureates of this prize. He encountered mountains of printed papers of unequal heights, separated by a narrow gorge leading to the desk, behind which Gérard was seated. The light was dim. At the time, Gérard was chairing the Claude Paoletti Prize Committee and this meeting took place in preparation for the award ceremony.

Gérard had the reputation of having read everything. He had certainly read our papers. The laureate was bombarded with incisive questions that went well beyond his papers and attested to both Gérard’s elephantine memory and his unsatiable curiosity. He did not feel that he had passed the test until Gérard asked him if his father was of Corsican descent, like Claude Paoletti. He will never know whether his surname contributed to his selection as the laureate.

Coming back to Paoletti and Orth, Paoletti suggested that Gérard should do his PhD studies in collaboration with the *Institut Pasteur*. He suggested that Gérard submit this project to Jacques Monod*.* The resulting meeting with Monod played a key role in Gérard’s career, leading him to resign from INRA in 1963. Monod received Gérard at the *Institut Pasteur* in November 1963. As Gérard recalls, “*Monod had not shown any obvious interest in the subject that C. Paoletti had suggested to me. He had told me about a recent article by Stanfield Rogers demonstrating that an L-arginase encoded by the viral genome was induced in cutaneous papillomas caused by the Shope papilloma virus in wild cottontail and, in experimental conditions, in domestic rabbits *[8]*. The possible malignant transformation of warts that this DNA virus induces under natural (cottontail rabbits) or experimental (domestic rabbits) conditions made the Shope virus the first paradigm for the study of viral carcinogenesis in mammals, thanks to the work of Peyton Rous and his colleagues. Unlike cottontail rabbit papillomas, domestic rabbit papillomas induced in experimental conditions display a repression of viral replication. Why did the induction of a cutaneous wart (and cancer) require a viral gene encoding an enzyme the cellular counterpart of which was involved in the last stage of hepatic ureogenesis?*”.

Jacques Monod invited Gérard to present an analytical seminar on this article in the presence of four future Nobel Prize winners: François Jacob, Salvatore Luria, André Lwoff, and Jacques Monod! Jacques Monod judged his seminar “*excellent*” and concluded: “*You have to work on that*”. This did not fail to impress Paoletti, who then suggested that Gérard should study arginase at Villejuif. Gérard recalled that “*the fact that a student of Jacques Monod, Jean-Pierre Changeux — who was then writing his famous thesis on the allosteric properties of L-threonine deaminase — was going to join the laboratory as one of the army of researchers working on this problem, was a determinant factor in my decision to take the plunge. And this, despite the difficulties of studying an enzyme so far removed from nucleases and despite the fact that papillomas were induced by a virus that did not grow* ex vivo*. The only source of the Shope virus was warts taken from living cottontail rabbits (*Sylvilagus floridanus*) from the banks of the Mississippi, as domestic rabbit papillomas only exceptionally produced virus! After my meeting with J. Monod, my scientific life was centered on cottontails or domestic rabbits for about ten years*.” Gérard also recalled that “*The collection of 34 rabbit statues in glass, crystal, porcelain or bronze that I have since amassed testifies to this! The faithful and affectionate friendship that binds us, Jean-Pierre and myself, also dates from this period*”.

Jean-Pierre Changeux, the eminent *Institut Pasteur* biochemist and neurobiologist, was Gérard’s only true friend from his own generation. Both were mentored or influenced by Lwoff, Monod, and Jacob. They shared an admiration for great minds and groundbreaking discoveries off the beaten track. They were elitist, placing scientific truth above all else, and they favored a Malthusian scientific effort. Moreover, despite differences in their political opinions, they spoke their minds freely, abhorring the politically correct discourse that progressively gained momentum in Western academia. Finally, both were patriots.

The results that Gérard obtained in collaboration with Françoise Vielle-Breitburd, under the supervision of Jean-Pierre Changeux, clearly demonstrated the cellular origin of the arginase of papillomas. Using partially purified enzyme preparations, they showed that the papilloma and liver arginases had similar kinetic and immunological properties, indicating that the genetic information for the tumor enzyme was probably carried by the rabbit genome [9]. These results, in opposition to those of Rogers, would have allowed Gérard to defend his thesis within a reasonable time frame. However, he did not defend his PhD, despite an agreement between Jacques Monod and Claude Paoletti. Jacques Monod had agreed to be a member of the PhD jury (Fig. 3). Gérard then recalled “*But I then decided to have myself as my only mentor, throughout my career, often at my own cost. This would not have been possible without the understanding, patience, and friendship of Claude Paoletti*”.

**Fig. 3:**
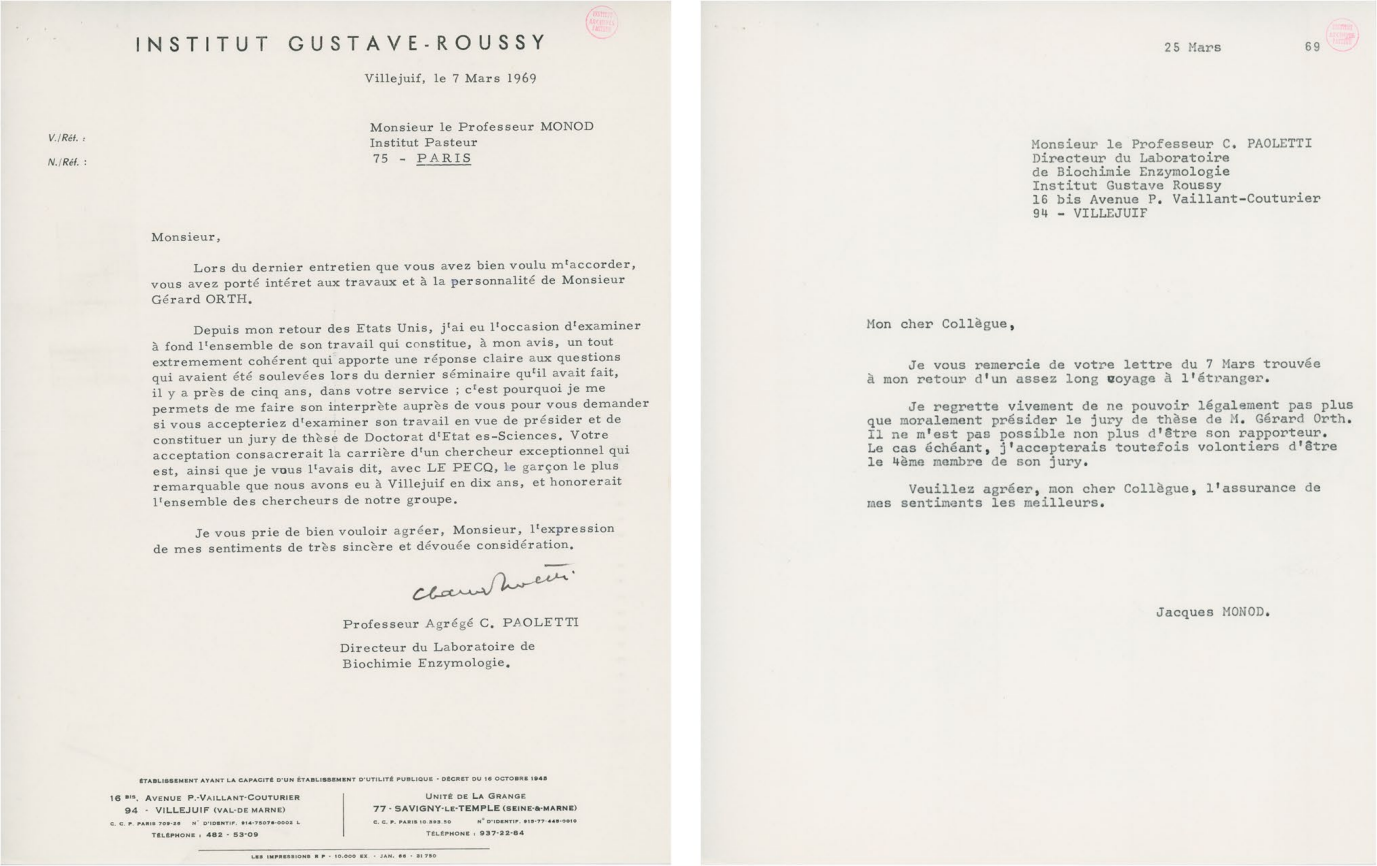
Letter of Claude Paoletti to Jacques Monod in 1969 about the PhD defense of Gérard Orth and the response of Jacques Monod (Pasteur Institute, (MON.COR.12))

In an attempt to reproduce the induction they had observed in vitro, Gérard and his collaborators obtained domestic rabbit keratinocytes with ultrastructural characteristics attesting to their epidermal origin [10]. The infection of cultures of these cells with the Shope virus did not lead to arginase induction. Only a modest increase in arginase activity was observed in papilloma cell cultures. Further collaborative work with Vielle-Breitburd led to the determination of the properties and subunit structure of highly purified rabbit liver arginase [11–13].

Such work would not have been possible without a long and fruitful collaboration with Odile Croissant, head of the electron microscopy laboratory of the Virus Department of the *Institut Pasteur*. One of the striking observations to emerge from this work was their demonstration of the induction of DNA synthesis in keratinizing keratinocytes from domestic rabbit warts, which produce virus only exceptionally, whereas no such DNA synthesis was observed in papillomas induced with dimethyl-benzanthracene. Rashad and Evans had reported similar observations [14]. One of the first applications of in situ hybridization to the study of viruses enabled Gérard and his coworkers to demonstrate that this DNA synthesis corresponded to cellular DNA, in the absence of viral DNA replication [15].

Gérard’s “non-defense” of his PhD thesis since 1966 led the CNRS to question his right to membership of this organization. As a result, Gérard, in a highly disturbed emotional state, devoted all his energy to another attempt at writing this thesis in 1971 and 1972, at home, in Sceaux. Gérard recalled that “*at the beginning of 1972, I submitted the manuscript, entitled ‘Analysis of viral genome expression during abortive or productive infection of the epidermal cell by the Shope papilloma virus’ to C. Paoletti, my PhD supervisor. The manuscript included two dedications, one referring to Louis Pasteur (‘If joy is in success, virtue is in effort’) and the other to André Lwoff (‘Despite the harsh disciplines it imposes, scientific research, as everyone knows, is part of playful activity’), in homage to two scientists who served as a reference in my moments of doubt. C. Paoletti had suggested minor modifications, and François Gros, the reviewer, and André Lwoff, member of the Jury accepted my manuscript: ‘I, the undersigned, André Lwoff, Honorary Professor at the Faculty of Sciences declare that I accept the thesis presented by Gérard Orth’, ‘I, the undersigned, Prof. F. Gros declare having examined Mr. Orth’s PhD thesis and having considered it acceptable for an imminent defense. F. Gros’ (April 17 1972)”*. However, Gérard recalled that “*After years of depression, I decided to turn the page and not defend this PhD, instead focusing on the study of the wart virus, hoping to find variations in the sequence of the viral genome that could account for clinical variations (localization of the lesion, outcome, epidemiology …)*” [16].

Indeed, Gérard had his ups and downs. One sentence he once whispered to us, not without hesitation, more than a decade after our first encounter, was “*Je ne m’aime pas*”. He didn’t mean “*I do not love myself*”. He felt that no-one should love themselves, because pride is one of the seven deadly sins. What he really meant is “*I do not like myself*”. At the crepuscule of his life, he had both remorse and regrets.

Nevertheless, Gérard could be both tactful and gracious. It was only after years of dinners at the *Dôme*, a seafood restaurant at Montparnasse, that he confessed to us that he hated seafood. After that, we crossed the Boulevard Montparnasse and dined at the *Rotonde*, his favorite place, or sometimes at the *Select* or the *Coupole*. He enjoyed these brasseries, where great writers and artists had gathered before World War II.

Having decided to focus on the wart virus, Gérard turned to human studies in 1973. His epiphany occurred in 1972, when he first heard of *epidermodysplasia verruciformis* in the article by Jablonska, S. Jakubowicz, K., Dabrowski, J. “Epidermodysplasia verruciformis as a model in studies on the role of papovaviruses in oncogenesis” published in Cancer Research in March 1972 [17]. This article highlighted the role of host genetic factors and immune factors (cellular immunity disorders possibly secondary to the chronicity of infection). Jablonska et al. then hypothesized that the papovavirus particles observed in benign lesions might play a role in the initiation of cell transformation specific to *epidermodysplasia verruciformis*. She also suggested that genetic factors were responsible for the chronicity of infection.

Another decisive moment came on December 15–16, 1975 (Medical aspects of human wart virus infection). Proceedings of a Fondation Mérieux seminar held in Lyon on December 15–16, 1975). This was the occasion of the first meeting of Gérard Orth with Stefania Jablonska. With hindsight, Gérard would say that Goret, Paoletti, Monod, Changeux, and Jablonska were the five people who counted the most in his scientific life.

Indeed, his true love was *epidermodysplasia verruciformis* [18–21]. Gérard was a great admirer of Stefania Jablonska, the head of the dermatology department in Warsaw, Poland. This was the Poland from before the fall of the Iron Curtain. Using hetero-inoculation, Jablonska had shown that *epidermodysplasia verruciformis* was triggered by a virus [22]. She and Gérard teamed up to identify and characterize this virus [23]. Although he had heard that Jablonska was not unfriendly to the communist regime, Gérard admired her, perhaps because of her extraordinary scientific achievements in such dire conditions, and perhaps because of her Jewish origins and decision to stay in Poland. He would not say more on this topic. He was particularly proud to have received the 1985 Robert Koch Prize with Jablonska, for which he gave his acceptance speech in Berlin in German. He was also elected a foreign member of the Polish Academy of Sciences in 1997.

Gérard was truly encyclopedic, and this could be utterly exhausting for anyone in his company. Mr Teste, a character created by Paul Valéry, would say that “*silliness is not my strength*”. It would be fair to say that concision was not Gérard’s strength. He could not provide a short answer to any question, in any circumstances. His e-mails, in particular, were Tolstoian, even though he had very little taste for anything Russian. His writing style was very formal, “*vieille France*” as the French would say. The last of the Mohicans.

Accordingly, his papers and his talks were extraordinarily long, complicated, obscure, and therefore difficult to follow. There was always an exception to an exception to an exception. He did not believe that there were any rules in biology. He was right, of course, to be a radical nominalist, because biology is not made for typologists, intellectuals who flourish in physics but tend to encumber biology and embarrass themselves by their perpetual search for the “laws of nature”. However, this attitude was not didactic, to say the least, and it certainly did not contribute to his popularity. But Gérard never considered himself to be in a popularity contest. Nobody understood the classification of human papillomaviruses like him. Maybe that was the problem with the classification that he helped to build.

Gérard recalled with a grin that he was called “*mein Bruder*” by Harald Zur Hausen; they held joint laboratory meetings for a time. Zur Hausen won the Nobel Prize for the development of a vaccine against oncogenic mucosal HPV, but it was Gérard who first showed that cutaneous HPV infections could be oncogenic in *epidermodysplasia verruciformis* patients [24], before the demonstration that genital HPV infection can underlie cervical cancer. Gérard tended to irritate his colleagues not only by always being right, but also by making it clear to them that he was right. However, this was not a question of pride. Instead, it attested to an absolute and sometimes excessive or exclusive respect for science.

Gérard was a giant in virology and a giant in immunology. He pioneered the field of papillomaviruses, discovered an entire genus of human papillomaviruses, the defective but oncogenic beta HPV, which cause disease in rare patients with *epidermodysplasia verruciformis* [16, 25–27]. As if being an extraordinary virologist were not sufficient, he discovered the first human genes underlying warts, in patients with *epidermodysplasia verruciformis* [28–30]. Who else can say that they have discovered both a microbial pathogen and the corresponding human genetic lesion? Gérard Orth had the unique technical versatility to be able to switch from viral identification to human linkage analysis, from sequencing viral genes to sequencing human genes, and the intelligence to understand that the microbe is merely a trigger that reveals an underlying, pre-existing condition of the host.

While the vast majority of microbiologists remained prisoners of Koch’s radical version of the germ theory, Gérard knew that microbes cause disease only rarely, in individuals in whom human genetic and immunological determinants of disease operate. He therefore decided to discover why some rare individuals develop flat warts and skin cancer triggered by the weakly virulent, defective beta-HPV he had discovered with Stefania Jablonska [16, 20].

The first time we heard the term “intrinsic immunity”, it came out of his mouth [18, 31–33]. Twenty-five years ago, he convinced us that *epidermodysplasia verruciformis* had to be due to a derailment of keratinocytic intrinsic immunity. At that time, keratinocytes had not even been considered as a possible branch of host defense. This idea gained ground when he found mutations underlying *epidermodysplasia verruciformis*.

Indeed, in 2002, Gérard published the breakthrough discovery that biallelic mutations of *EVER1* or *EVER2* underlie a significant proportion of cases of *epidermodysplasia verruciformis* [29]. Luckily, the two genes are located in tandem, accounting for the single linkage peak. This discovery was the second elucidation of the molecular genetic basis a known Mendelian infection, of which five were known at the time [34, 35]. Nicolas Ramoz, the PhD student who performed this work, defended his thesis at the *Institut Pasteur*, where it attracted little attention. One of us (JLC) was a member of the jury. Gérard was forced to retire soon after this breakthrough, whilst still in his prime, attesting to the nonsensical and counterproductive rigidity of the employment rules in French academia.

Gérard was continually surprised and dismayed by the decline of the scientific community of France and the *Institut Pasteur*. He could not help wondering how Pasteur, an institute as richly endowed as some of the wealthiest American institutions, a unique feature in the European research landscape, had produced so little since the late 1960s, and why even when it did produce results of interest, it was often by accident. How could an institute with so much money produce so little science? This was one of his recurrent questions. The discovery of HIV was one of his favorite examples. Gérard loved quoting François Jacob allegedly saying during a meeting: “*People say Montagnier discovered a virus; I think a virus discovered Montagnier*”.

Gérard was also particularly fond of another anecdote. A colleague of his at *Institut Pasteur* had doubts about the scientific quality of a younger French scientist because of his many publications in prestigious journals. Gérard was fond of Johan Sebastian Bach and had read (in German) the essay about Bach written by his Alsatian compatriot Albert Schweitzer (the physician, Nobel Prize winner, who had spent his professional life in the tropical forest of Gabon, was born in German-occupied Alsace, and was a relative of Jean-Paul Sartre — who declined the Nobel Prize). Distraught by the insanity of this concern, Gérard would say, tongue in cheek: “*My colleague would doubt Bach’s genius because Bach composed a masterpiece almost every week*”.

Gérard grew increasingly tired of having to tolerate the mediocrity around him. He became misanthropic and was further weakened by his myelodysplasia. In 2022, he was unable to travel to Newport Beach to receive the Thomas A. Waldmann Award for excellence in the field of human inborn errors of immunity, which he so richly deserved, in person from Sudhir Gupta. He gave his speech by video link, and he looked exhausted. In previous years, Gérard not only frequently came to visit our laboratory in Paris, but also hopped on a plane to spend some time with us in New York.

When he retired in 2003, Gérard joined our laboratory as a consultant. He even made us his “*légataire universel*”. He gave us all his samples, contacts, patients, archives, and, more importantly, all his thoughts. The CIB1 saga that followed was particularly exciting [36]. After having found mutations of T-cell genes in patients with an “atypical” form of epidermodysplasia verruciformis, we finally found mutations of a new gene in patients with a “typical” form of *epidermodysplasia verruciformis*. These patients did not suffer from other infections. Jill de Jong, in our laboratory, connected the dots and found that CIB1 forms a multimer with EVER1 and EVER2. Some of these patients were even of particular importance to Gérard, including one originally described by Wilhelm Lutz in Basel [37] and one family from Algeria that Gérard had followed for decades. Gérard was content — a rare state of mind for him.

We published nine papers together. Our next paper on *epidermodysplasia verruciformis* will be dedicated to him.

Gérard died while working on a paper retracing his career that he had promised for the *Journal of Clinical Immunology*. We found only three successive versions of a draft retracing the first two decades of his career on his computer. Many of the quotes in this obituary were taken from these documents, which also inspired this obituary.

Even after a glass of wine, or two (or even three), or with a cigar, Gérard would only sparingly and timidly share anecdotes about his private life. He loved the impressionists and Hansi (Fig. 4A). Hansi (1863–1951) was an Alsatian illustrator and French patriot. He was particularly fond of Pissarro (1930–1903), as many paintings of his are of Pontoise and Saint Ouen l’Aumône, where Gérard grew up (*Les bords de l’Oise près de Pontoise; Bords de l’Oise; Usine à Saint-Ouen l’Aumône 1873. La crue de l’Oise; La sucrerie au bord de l’Oise; Usine près de Pontoise; Le Quai du Pothuis à Pontoise*) (Fig. 4B). These paintings opened a window onto his youth.

**Fig. 4:**
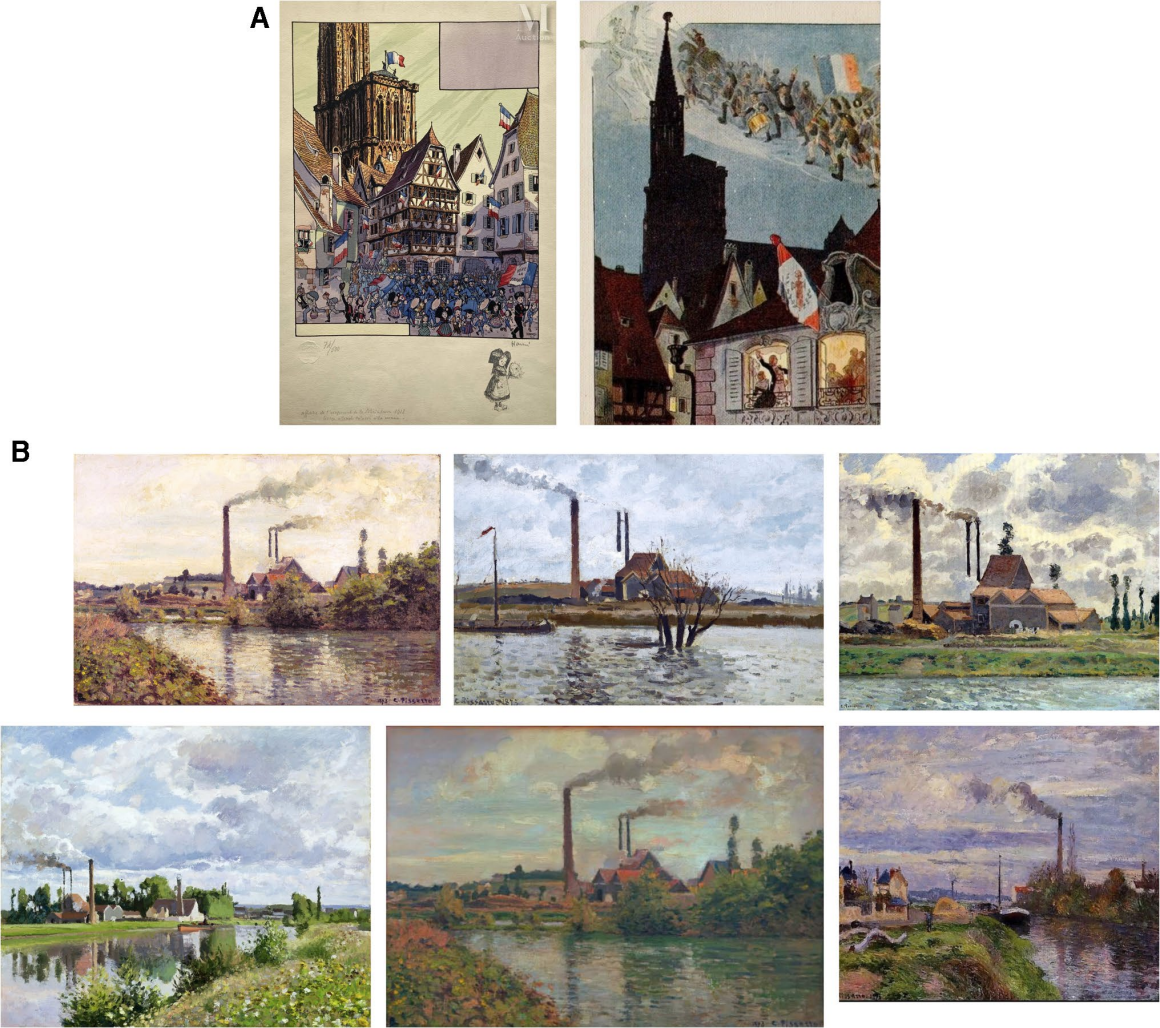
Hansi and Pissarro paintings at Pontoise from 1867 to 1878. **A** Selection of 2 paintings from Hansi. **B** Selection of 6 paintings of Pissarro on “L’usine de Pontoise”

Gérard never married and did not have children. This was a source of regret to him. He bitterly regretted his loneliness, which increased when his laboratory closed and was mitigated only partially by his frequent conversations with Jean-Pierre Changeux, his work in our laboratory, and our dinners at the *Rotonde*. However, he had no regrets about having devoted so much time to rabbits, papillomaviruses, *epidermodysplasia verruciformis*, and intrinsic immunity (Fig. 5). He was also glad to have been inspired by five extraordinary mentors and colleagues.

**Fig. 5:**
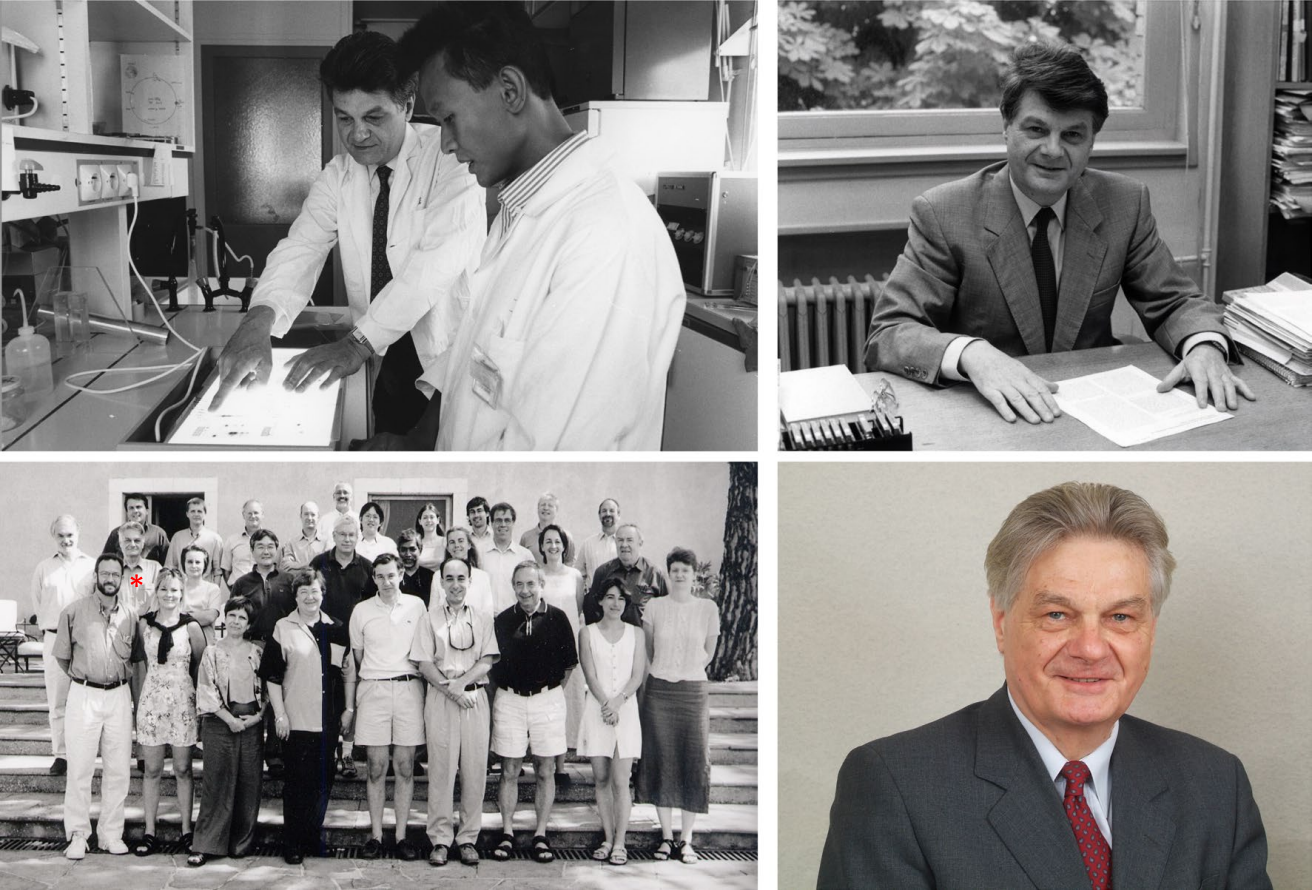
Top left: Around 1990, Gérard Orth and a researcher analyzing an electrophoresis (photography of Béatrice de Cougny, Pasteur Institute). Top right: Around 1990, Gérard Orth in his office. Bottom left: In 2002, Gérard Orth attending a workshop on host genetics of infectious diseases organized by Jean-Laurent Casanova at the Fondation des Treilles (Gérard Orth is indicated by the red cross). Bottom Right: A portrait of Gérard Orth around 2010

Gérard did not like himself, but we loved him. He will be sorely missed.

## Data Availability

No datasets were generated or analysed during the current study.

## References

[1] Goret P, Mornet P, Gilbert Y, Pilet C, Orth G (1960). Research on crossed immunization “Carre’s disease-bovine plague” in the rabbit. Ann Inst Pasteur (Paris).

[2] Orth G (2022). Pasteur and the veterinarians. C R Biol.

[3] Lepine P (1957). Present status of vaccination against poliomyelitis. Rev Pathol Gen Physiol Clin.

[4] Sabin AB (1957). Present status of attenuated live virus poliomyelitis vaccine. Bull N Y Acad Med.

[5] Salk JE (1957). Poliomyelitis vaccination in the fall of 1956.. Am J Public Health Nations Health.

[6] Lepine P (1954). Determination of production of in vitro cultures of poliomyelitis virus by evaluating the numerical relationship between cellule-host and free virus. C R Hebd Seances Acad Sci.

[7] Orth G, Atanasiu P, Boiron M, Rebiere JP, Paoletti C (1964). Infectious and oncogenic effect of DNA extracted from cells infected with polyoma virus. Proc Soc Exp Biol Med.

[8] Rogers S, Moore M (1963). Studies of the mechanism of action of the Shope rabbit papilloma virus. I. Concerning the nature of the induction of arginase in the infected cells. J Exp Med..

[9] Orth G, Vielle F, Changeux JP (1967). On the arginase of the Shope papillomas. Virology.

[10] Orth G, Croissant O (1967). Morphologic and ultrastructural characteristics of cells of the epidermis of adult rabbits in tissue culture. C R Hebd Seances Acad Sci D.

[11] Vielle-Breitburd F, Orth G (1972). Rabbit liver L-arginase. Purification, properties, and subunit structure. J Biol Chem..

[12] Orth G, Jibard N, Vielle-Breitburd F (1971). Purification and properties of L-arginase of papillomas induced with the rabbit papilloma virus. C R Hebd Seances Acad Sci D.

[13] Vielle-Breitburd F, Orth G (1971). Structure of rabbit hepatic L-arginase. C R Hebd Seances Acad Sci D.

[14] Rashad AL, Evans CA (1967). A difference in sites of DNA synthesis in virus-induced (Shope) and in chemically induced epidermal tumors of rabbit skin. Cancer Res.

[15] Orth G, Jeanteur P, Croissant O (1971). Evidence for and localization of vegetative viral DNA replication by autoradiographic detection of RNA-DNA hybrids in sections of tumors induced by Shope papilloma virus. Proc Natl Acad Sci U S A.

[16] Orth G, Jablonska S, Breitburd F, Favre M, Croissant O (1978). The human papillomaviruses. Bull Cancer.

[17] Jablonska S, Dabrowski J, Jakubowicz K (1972). Epidermodysplasia verruciformis as a model in studies on the role of papovaviruses in oncogenesis. Cancer Res.

[18] Orth G (2006). Genetics of epidermodysplasia verruciformis: insights into host defense against papillomaviruses. Semin Immunol.

[19] Orth G (2008). Host defenses against human papillomaviruses: lessons from epidermodysplasia verruciformis. Curr Top Microbiol Immunol.

[20] Pass F, Reissig M, Shah KV, Eisinger M, Orth G (1977). Identification of an immunologically distinct papillomavirus from lesions of epidermodysplasia verruciformis. J Natl Cancer Inst.

[21] Jablonska S, Orth G, Jarzabek-Chorzelska M, Rzesa G, Obalek S, Glinski W (1978). Immunological studies in epidermodysplasia verruciformis. Bull Cancer.

[22] Jablonska S, Milewski B (1957). Information on epidermodysplasia verruciformis Lewandowsky-Lutz; positive results of auto- and heteroinoculation. Dermatologica.

[23] Orth G, Jablonska S, Favre M, Croissant O, Jarzabek-Chorzelska M, Rzesa G (1978). Characterization of two types of human papillomaviruses in lesions of epidermodysplasia verruciformis. Proc Natl Acad Sci U S A.

[24] Orth G, Jablonska S, Jarzabek-Chorzelska M, Rzesa G, Obalek S, Favre M, et al. Characteristics of the lesions and risk of malignant conversion as related to the type of the human papillomavirus involved in epidermodysplasia verruciformis. Cancer Res. 1979;39:1074–82. 218721

[25] Deau MC, Favre M, Orth G (1991). Genetic heterogeneity among human papillomaviruses (HPV) associated with epidermodysplasia verruciformis: evidence for multiple allelic forms of HPV5 and HPV8 E6 genes. Virology.

[26] Favre M, Orth G, Croissant O, Yaniv M (1975). Human papillomavirus DNA: physical map. Proc Natl Acad Sci U S A.

[27] Orth G, Favre M, Croissant O (1977). Characterization of a new type of human papillomavirus that causes skin warts. J Virol.

[28] Ramoz N, Rueda LA, Bouadjar B, Favre M, Orth G (1999). A susceptibility locus for epidermodysplasia verruciformis, an abnormal predisposition to infection with the oncogenic human papillomavirus type 5, maps to chromosome 17qter in a region containing a psoriasis locus. J Invest Dermatol.

[29] Ramoz N, Rueda LA, Bouadjar B, Montoya LS, Orth G, Favre M (2002). Mutations in two adjacent novel genes are associated with epidermodysplasia verruciformis. Nat Genet.

[30] Ramoz N, Taieb A, Rueda LA, Montoya LS, Bouadjar B, Favre M (2000). Evidence for a nonallelic heterogeneity of epidermodysplasia verruciformis with two susceptibility loci mapped to chromosome regions 2p21-p24 and 17q25. J Invest Dermatol.

[31] Bieniasz PD (2004). Intrinsic immunity: a front-line defense against viral attack. Nat Immunol.

[32] Yan N, Chen ZJ (2012). Intrinsic antiviral immunity. Nat Immunol.

[33] Randow F, MacMicking JD, James LC (2013). Cellular self-defense: how cell-autonomous immunity protects against pathogens. Science.

[34] Casanova JL (2023). From second thoughts on the germ theory to a full-blown host theory. Proc Natl Acad Sci U S A.

[35] Casanova JL, Abel L (2022). From rare disorders of immunity to common determinants of infection: following the mechanistic thread. Cell.

[36] de Jong SJ, Crequer A, Matos I, Hum D, Gunasekharan V, Lorenzo L (2018). The human CIB1-EVER1-EVER2 complex governs keratinocyte-intrinsic immunity to beta-papillomaviruses. J Exp Med.

[37] Lutz W (1946). About verruciform epidermodysplasia. Dermatologica.

